# Molecular Identification of Pathogenic Free-Living Amoeba from Household Biofilm Samples in Iran: A Risk Factor for *Acanthamoeba* Keratitis

**DOI:** 10.3390/microorganisms9102098

**Published:** 2021-10-05

**Authors:** Maryam Norouzi, Reza Saberi, Maryam Niyyati, Jacob Lorenzo-Morales, Hamed Mirjalali, Marziye Fatemi, Ehsan Javanmard, Seyed Ahmad Karamati

**Affiliations:** 1 Department of Medical Parasitology and Mycology, School of Medicine, Shahid Beheshti University of Medical Sciences, Tehran 11369, Iran; mariam.1356@yahoo.com (M.N.); marziye.fatemi1994@gmail.com (M.F.); 2 Department of Medical Parasitology, School of Medicine, Mazandaran University of Medical Sciences, Sari 48175, Iran; reza.sab.68@gmail.com; 3 Infectious Diseases and Tropical Medicine Research Center, Shahid Beheshti University of Medical Sciences, Tehran 11369, Iran; 4 Instituto Universitario de Enfermedades Tropicales y Salud Pública de Canarias (IUETSPC), Universidad de La Laguna (ULL), Avda. Astrofísico Fco. Sánchez s/n, 38203 San Cristóbal de La Laguna, Spain; jmlorenz@ull.edu.es; 5 Red de Investigación Cooperativa en Enfermedades Tropicales (RICET), 28006 Madrid, Spain; 6 Departamento de Obstetricia y Ginecología, Pediatría, Medicina Preventiva y Salud Pública, Toxicología, Medicina Legal y Forense y Parasitología, Universidad de La Laguna (ULL), 38200 San Cristóbal de La Laguna, Spain; 7 Foodborne and Waterborne Diseases Research Center, Research Institute for Gastroenterology and Liver Diseases, Shahid Beheshti University of Medical Sciences, Tehran 11369, Iran; hamed_mirjalali@hotmail.com; 8 Department of Medical Parasitology and Mycology, School of Public Health, Tehran University of Medical Sciences, Tehran 14114, Iran; ehsan_javan1990@yahoo.com; 9 Department of Medical Parasitology and Mycology, Faculty of Medicine, Tehran Medical Sciences, Islamic Azad University, Tehran 57169, Iran; sa.karamati@yahoo.com

**Keywords:** free-living amoeba, biofilm, genotyping, Iran

## Abstract

Free-living amoeba (FLA) are ubiquitously distributed in the environment. However, they are also the causative agents of opportunistic infections in humans and other animals. A biofilm comprises any syntrophic consortium of microorganisms in which cells stick to each other and often also to a surface. Moreover, FLA have been detected in various biofilms around the world. Therefore, the present study aimed to check for presence of FLA in samples from household biofilms in Iran and to characterize them at the molecular level. A total of 69 biofilm samples collected from showerheads, kitchen areas, and bathroom sinks were analyzed. Positive samples for FLA were characterized at the morphological and molecular levels. Furthermore, the results of morphology analysis indicated that 26.08% (18/69) of biofilm samples were positive for *Acanthamoeba* spp., *Vermamoeba* genus, and Vahlkampfiids. According to sequence analysis, five strains of *Acanthamoeba* isolates related to the T4 genotype and two strains belonged to the T2 genotype. In addition, the pathogenic potential of *Acanthamoeba*-positive isolates was conducted using the tolerance ability test. The results of BLASTn of *Vermamoeba* sequences were similar to what was expected for *Vermamoeba vermiformis*. The above-mentioned reasons revealed that the relative high contamination of household biofilm samples with FLA may pose a risk for people using soft contact lenses and for patients with traumatic cataract. Our finding proposes that filtration should be performed in shower heads and indicates the need to monitor people at increased risk of *Acanthamoeba* keratitis.

## 1. Introduction

Free-living amoeba (FLA) are widely distributed in a variety of ecological and environmental sources, such as soil, dust, recreational and drinking water, and medical equipment [1,2,3]. Among them, four genera/species have been associated with human disease: *Acanthamoeba* spp., *Naegleria fowleri*, *Balamuthia mandrillaris*, and *Sappinia pedata* [4]. These pathogenic protozoa can complete their life cycles in the environment without entering human or animal hosts [5]. Furthermore, the role of *Vermamoeba vermiformis* as a pathogenic parasite of humans is still unknown [6]. There are several studies regarding *V. vermiformis* as an environmental contaminant, but there are also reports of eye infections [7,8].

A biofilm constitutes an assemblage of microorganisms in which cells adhere to each other and often to an abiotic or biotic surface [9]. In addition, it could work as an adaptable system to environmental conditions and could also play a crucial role in the protection of pathogenic agents in harsh conditions [10]. Hence, biofilm formation in intraocular and contact lenses, coronary stents, intravascular catheters, urinary catheters, and neurosurgical ventricular shunts has accelerated the necessity of biofilm research [11,12]. Meanwhile, the development of household biofilms should not be neglected since they can be a threat to inhabitants [13]. According to the literature review, FLA and notably *Acanthamoeba* spp. have been detected in various biofilms around the world [14,15,16]. 

*Acanthamoeba* spp. is a common FLA that presents two stages in its life cycle: the metabolically active trophozoite, and a dormant, stress-resistant cyst [17]. *Acanthamoeba* spp. can cause two well-defined diseases, including amoebic keratitis and granulomatous amoebic encephalitis [18]. *Acanthamoeba* keratitis (AK) is a rare sight-threatening infection of the cornea typically occurring in healthy contact-lens wearers [19]. Based on the complete sequence of the 18S rRNA gene, the genus of *Acanthamoeba* is divided into 23 different genotypes (T1–T23) [20]. The previous evidence showed that 12 of the genotypes (T2, T3, T4, T5, T6, T7, T9, T10, T11, T12, T13, and T15) can cause AK, but the most common genotype in clinical infections is T4 [21]. Furthermore, household biofilms are an important reservoir for *Acanthamoeba* spp. and bacterial endosymbionts in other FLA, which can cause amoebic keratitis in high-risk people during cleaning of lenses, exposure to ocular trauma, and showering while wearing contact lenses [16].

The occurrence of *Acanthamoeba* genotypes in biofilm sources in Iran is not widely studied [22,23]. Thus, our work aimed to identify FLA in household biofilms in selected houses in Iran at the morphological and molecular level and also to assess the pathogenic potential of the isolated *Acanthamoeba* strains.

## 2. Materials and Methods

### 2.1. Sampling and Culture of Amoebae

Sixty-nine biofilm samples from showerheads (*n* = 35), kitchen sinks (*n* = 20), and bathroom sinks (*n* = 14) were collected in 23 houses in Tehran Province, capital of Iran. The samples were collected by swabbing the interior surface of showerheads and sinks. After sampling, for the cultivation of amoeba, the sterile swabs were placed into 10 mL of sterile phosphate-buffered saline (PBS) and the obtained suspension was also processed by membrane filtration. After that, membrane filters were cultured onto 1.5% non-nutrient agar (Difco, Sparks, MD, USA) enriched with TYM (trypticase, yeast, and maltose). Plates were sealed with parafilm and incubated at room temperature with daily monitorization for up to a month.

### 2.2. Morphological Detection of FLA

According to Page’s description (Page, 1988), FLA were identified using morphological features at the genus level. The keys to morphological detection of *Acanthamoeba* trophozoites are listed as irregular shapes, acanthopodia, food vacuole, and a size of 25 μm. *Acanthamoeba* cysts are known as double-walled, hexagonal, spherical, polygonal, or star-like with diameters of 10–15 μm. *Vermamoeba* trophozoites are characterized as featuring division by binary fission, typical worm-shaped or slug-like (elongated, cylindrical) shapes, unclear nucleus, food vacuoles, and motility with sizes between 10 and 15 μm, whereas *Vermamoeba* cysts are recognized by double-walled, spherical, and round shapes with a single central nucleus and chromatin bodies. In addition, the diagnostic keys for vahlkampfiid amoebae trophozoites include the amoebic shape, a nucleus with a large central karyosome, a contractile vacuole, and size ranges of 10–35 μm. Finally, Vahlkampfiid amoebae cysts are identified by a thick wall, central nucleus, and variable size ranging from 10 to 12 μm.

### 2.3. Molecular Detection of FLA

#### 2.3.1. DNA Extraction and PCR Amplification

The amoebae from positive plates, at a density of 1 × 10^5^ parasites/mL, were washed three times with PBS (pH 7.2). After that, DNA was extracted from amoebae suspension using a nucleic acid extraction kit (Favorgen Biotech, Taiwan) and modified phenol–chloroform methods.

A PCR assay was performed using genus-specific primers, including JDP1: 5′ GGCCCAGATCGTTTACCGTGAA 3′ and JDP2: 5′ TCTCACAAGCTGCTAGGGAGTCA 3′ that amplified a 460 bp partial sequence of the 18s rRNA gene in *Acanthamoeba* species. In addition, to amplify a 700 bp fragment of the 18srRNA gene in *Vermamoeba*, a set of NA1: 5′ GCTCCAATAGCGTATATTAA 3′ and NA2: 5′ AGAAAGAGCTATCAATCTGT 3′ primers was used. The PCR amplification was carried out in a 25 µL PCR reaction mixture consisting of 12.5 µL of master mix (Ampliqon, Odense, Denmark), 1 µL each of forward and reverse primers, and 3 µL of DNA templet. The final volume was made with 25 µL by the addition of nuclease-free water. The PCR cycle profile was as follows: a primary denaturing step at 94 °C for 3 min, followed by 35 cycles of denaturation at 94 °C for 35 s; the annealing cycle at 56 °C and 50 °C for JDP and NA primers, respectively, for 45 s; and the extension cycle at 72 °C for 1 min. The final extension cycle was performed at 72 °C for 5 min. The 3–5 μL of PCR products were confirmed by visualization on 1.5% agarose gel (Invitrogen, Life Technologies GmbH, Leipzig, Germany) stained with SYBR-safe gel stain (Thermo Fisher Scientific, Waltham, MA, USA).

#### 2.3.2. Sequencing, Homology, and Phylogenetic Analysis

PCR products were sequenced in both directions using an ABI PrismTM 3730 Genetic Analyzer (Applied Biosystems, Foster City, CA, USA) by the Macrogen Company (Seoul, South Korea). In order to conduct homology analysis, DNA sequencing using the Basic Local Alignment Search Tool (BLAST) from the National Center for Biotechnology Information homepage (NCBI) was blasted with reference sequences available in the GenBank database. Nucleotide sequence data were deposited to the GenBank using BankIt under the accession number MZ458337-MZ458347.

### 2.4. Thermotolerance and Osmotolerance Assay

Based on previous studies, the pathogenesis tests were performed on the positive plates for the *Acanthamoeba* genus. Briefly, amoebae were exposed to heat (37 and 42 °C) for about a week to establish thermotolerance. In addition, osmotolerance of the amoeba was studied by using D-Mannitol (Merck, Darmstadt, Germany) at different molarities (0.5 and 1 M).

## 3. Results

### 3.1. Identification of FLA Based on the Morphological Characteristics

Based on the morphological characteristics, out of 69 biofilm samples, 18 (26.08%) were positive for FLA. From a total of 23 houses, 14 (60.86%) houses were positive for at least one type of FLA. It is also important to note that 10 (28.5%) showerhead, five (25%) kitchen sink, and three (21.4%) bathroom sink samples were positive for FLA. Among the positive samples, 11 (61.1%) were for *Acanthamoeba* spp., three (16.7%) were for *Vermamoeba*, three (16.7%) were for *Vahlkampfiids*, and one (5.5%) presented both *Acanthamoeba* spp. and *Vermamoeba* sp.

### 3.2. Identification of FLA Based on the Molecular Characteristics

Despite several attempts, due to fungal and bacterial contaminations, seven strains were lost during the purification process. Therefore, DNA was extracted from 11 samples and subjected to molecular identification. Based on the amplification of the 460 and 700 bp products in the amplification of the 18s rRNA gene, eight (72.7%) and three (27.3%) samples were positive for *Acanthamoeba* spp. and *Vermamoeba* sp., respectively. The DNA sequence analysis of the *Acanthamoeba*-positive cultures showed that five strains (A2, A3, A4, A6, and A10) belonged to the T4 genotype, and two (A7 and A9) were identified as T2 genotype members (Table 1). The isolated T2 genotypes from clinical and environmental studies in Iran are shown in Table 2. The A1 strain, which was identified as *Acanthamoeba* spp. at the morphological level, due to sequencing issues was not assigned any genotype. On the other hand, BLASTn of *Vermamoeba* sequences showed a 97–100% homology with registered species of *V. vermiformis* (Table 1). It is also important to mention that among the samples of houses that were positive for FLA (*Acanthamoeba* spp. and *V. vermiformis*), four residents had a history of trauma, cataract, and astigmatism surgery (Table 3).

### 3.3. Results of Tolerance Assays

According to the pathogenicity test, if *Acanthamoeba* spp. can grow at high temperatures (42 °C) and high osmolarity (1 M), it is considered a potentially pathogenic amoeba. Our assays revealed that six *Acanthamoeba* strains (A2, A3, A6, A9, and A10) could be considered highly pathogenic. However, two strains (A4 and A7) displayed growth at 37 °C and 0.5 M osmolarity and were not considered pathogenic (Table 4).

## 4. Discussion

Free-living amoebae are microorganisms of interest from both the environmental and clinical point of view [34]. Moreover, these amoebae can act as a Trojan horse of pathogenic bacteria and fungi such as *Pseudomonas aeruginosa*, *Legionella* spp., *Escherichia coli*, and *Cryptococcus neoformans* [35]. Furthermore, bacterial endosymbionts have been previously reported to enhance their survival and cause acute and highly destructive keratitis i.e., *Pseudomonas* species [36].

In recent years, the number of studies on FLA and especially *Acanthamoeba* spp. has risen in parallel to the increase in reported cases of AK worldwide [37]. Therefore, identifying the habitats of these amoebae and determining their genotype and pathogenic potential should be highly considered.

Furthermore, the *Acanthamoeba* genus has been found in a wide variety of natural habitats, including biofilms. Previously, the relationship between *Acanthamoeba* species and biofilms in different places (hospital, aquatic, ophthalmic, and dental environments) revealed that amoebae use biofilms for their survival and the dissemination of pathogens in nature [16].

In our study, from 69 biofilm samples collected from showerheads and kitchen and bathroom sinks, 18 were positive for FLA. Among the positive samples, *Acanthamoeba* strains were more prevalent than other FLA, which is in accordance with previous studies [16,22,38]. A previous study in Iran reported the presence of FLA in biofilm and dust from hospital wards hosting immunodeficient patients. Moreover, *Acanthamoeba* spp., *Hartmannella*, and *Vahlkampfia* were detected, which is consistent with our results [22]. In another study in Iran, biofilms from six indoor recreational water centers in Iran were positive for *Acanthamoeba* genotypes T3 and T4 [23].

Another study reported the contamination of bathroom sink water by *Acanthamoeba* in Hong Kong, and this contamination was estimated to be 10%, of which isolates were observed in samples received from urban areas and older buildings [39]. The presence of *Acanthamoeba* spp. in showerheads and kitchen and bathroom sinks is an important public health concern that helps us to understand the potential risk of developing AK in contact-lens wearers. In this study, four homeowners who were positive for FLA had a history of surgery, which could be considered a risk factor for them. As a result, hygiene management around sinks and showerheads should be encouraged. Previous studies support that water exposure of contact lenses is an important risk factor for AK [40]. The fast flow of water through shower heads may have ousted *Acanthamoeba*, which allows the amoeba to enter the eye from the water source [40]. In a study conducted by Muchesa et al., out of 97 water and biofilm samples isolated from tap water, dry swabs, and shower water, 90% of the samples were positive for *Acanthamoeba* spp., *Vermamoeba*, and *Naegleria* [38]. A study by Stockman et al. reported a high prevalence (79%) of *Acanthamoeba* spp. and other FLA in the water of bathroom showerheads in two counties of Ohio, USA [41]. It should be noted that the detection of amoeba was higher in swabs of biofilm compared to only collected water, which supports the method of collecting samples of our study [41].

*Acanthamoeba* genotyping provides valuable information, such as virulence, drug susceptibility, and clinical aspects. In this study, T2 and T4 genotypes were identified based on homology analysis of the 18S rRNA gene, and the data are comparable to those obtained by other researchers [42]. *Acanthamoeba* genotype T4 is the most predominant environmental genotype and more than 90% of AK cases have been associated with this genotype worldwide [43]. Moreover, our study represents the first report of the T2 genotype from biofilm samples in Iran.

In Table 2, we summarized previous environmental sources of the T2 genotype in Iran. According to our literature review, 11 studies in Iran have reported on genotype T2, which mostly belong to environmental samples. However, in the study by Maghsoud et al., T2 was isolated from the contact lenses of AK patients [24]. *Acanthamoeba* genotype T2 is present in different sources and places and causes AK.

## 5. Conclusions

In conclusion, the relative high FLA contamination of household biofilm samples reported in this study may pose a risk for people using soft contact lenses and for patients with traumatic cataracts. Thus, spout filters and attention to personal hygiene to avoid encountering *Acanthamoeba* cysts in contact-lens wearers is highly recommended in the studied area.

## Figures and Tables

**Table 1:** Data regarding the positive samples of FLA using molecular identification.

Strain Code	Source	Isolated Amoebae	Genotype/Species	Identity/Query Coverage(%)	Accession Number	Similar Accession Numbers	Sources/Country of Reference
A1	Showerhead	*Acanthamoeba*	NA	97/98.7	MZ458337	NA	NA
A2	Bathroom sink	*Acanthamoeba*	T4	97/99.2	MZ458338	KU356844.1 KR074219.1	Water/Iran
A3	Bathroom sink	*Acanthamoeba*	T4	95/99.5	MZ458339	KF924601.1	Danube River sediments
A4	Showerhead	*Acanthamoeba*	T4	97/99.5	MZ458340	KU356850.1	Water Iran
A6	Showerhead	*Acanthamoeba*	T4	93/96.5	MZ458341	AY173005.1/ MT378227	Marine sediment Korea/water and keratitis Iran
A7	Bathroom sink	*Acanthamoeba*	T2/ *palestinensis*	91/91	MZ458342	MH678804.1/ MN227255.1	Water Spain/ Farming land Iran
A9	Showerhead	*Acanthamoeba*	T2/ *palestinensis*	99/96	MZ458343	MN227255.1	Farming land Iran
A10	Kitchen sink	*Acanthamoeba*	T4	96/99	MZ458344	LC604811.1	Bronchoalveoal lavage Iran
H5	Showerhead	*Vermamoeba*	*vermiformis*	99/99.8	MZ458347	MK418871.1	Wound in the upper eye Germany
H8	Kitchen sink	*Vermamoeba*	*vermiformis*	100/100	MZ458345	MK418871.1	Wound in the upper eye Germany
H10	Showerhead	*Vermamoeba*	*vermiformis*	99/97.1	MZ458346	MT292608.1	Water Iran

Abbreviations: NA, not applicable.

**Table 2:** Several studies that reported *Acanthamoeba* genotype T2 from different samples and provinces of Iran.

Author	Year	Location	No. T2 Genotype	Source	Reference
Maghsood et al.	2005	Hamedan	7	Fountain pool water	[24]
Maghsood et al.	2005	Iran	3	Contact lens	[24]
Niyyat et al.	2009	Tehran	1	Pool water	[25]
Rahdar et al.	2012	Khuzestan	1	Agricultural water	[26]
Hooshyar et al.	2013	Qazvin	3	Stagnant water	[27]
Shokri et al.	2016	Mazandaran	3	Farm water and fish pool	[28]
Niyyati et al.	2016	Ilam	1	Geothermal rivers	[29]
Golestani et al.	2018	Kashan	1	Hospital dust	[30]
Pazoki et al.	2019	Tehran	2	Farming land Recreational centers	[7]
Saberi et al.	2019	Ilam	4	Dust	[31]
Abdi et al.	2020	West Azerbaijan	1	Surface Water	[32]
Mahmoodi et al.	2021	Guilan	5	Caspian seawater	[33]
Mahmoodi et al.	2021	Guilan	7	Hospital ward dust	[33]

**Table 3:** Demographic and risk factors of people living in houses positive for FLA.

Isolate Code	Isolated Amoebae	Accession Number	Age	Gender	Occupation	Literacy	Surgery Reason	Risk Factor
A9	*Acanthamoeba* T2	MZ458343	72	Male	Gardener	Diploma	Trauma	Diabetes
A2	*Acanthamoeba* T4	MZ458338	35	Male	University student	University student	Trauma	_
A4	*Acanthamoeba* T4	MZ458340	80	Female	Housewife	High school	Cataract	Diabetes
H8	*V. vermiformis*	MZ458345	38	Female	Makeup artist	University degree	Astigmatism	_

**Table 4:** Data regarding the pathogenicity of *Acanthamoeba* spp. using a tolerance test.

Strain Code	Genotype	Osmotolerance (0.5/1 M)	Thermotolerance (37/42)	House Code (with High-Risk Residents)
A2	T4	+/+	+/+	H3
A3	T4	+/+	+/+	−
A4	T4	+/−	+/−	H10
A6	T4	+/+	+/+	−
A7	T2	+/−	+/−	−
A9	T2	+/+	+/+	H12
A10	T4	+/+	+/+	−

## Data Availability

The data associated with this manuscript are included in the article.

## Institutional Board Statement

All procedures performed in this study were in accordance with the ethical standards (IR.SBMU.RETECH.REC.1399.702) released by the Ethical Review Committee of Shahid Beheshti University of Medical Sciences, Tehran, Iran.
